# Effect of Apical Preparation Size and Preparation Taper on Smear Layer Removal Using Two Different Irrigation Needles: A Scanning Electron Microscopy Study

**DOI:** 10.1055/s-0044-1791682

**Published:** 2024-12-30

**Authors:** Rania Lebbos, Naji Kharouf, Deepak Mehta, Jamal Jabr, Cynthia Kamel, Roula El Hachem, Youssef Haikel, Marc Krikor Kaloustian

**Affiliations:** 1 Department of Endodontics, Faculty of Dental Medicine, Saint Joseph University of Beirut, Beirut, Lebanon; 2 Department of Biomaterials and Bioengineering, INSERM UMR_S 1121, Strasbourg University, Strasbourg, France; 3 Department of Endodontics and Conservative Dentistry, Faculty of Dental Medicine, Strasbourg University, Strasbourg, France; 4 Department of Cariology, Saveetha Dental College and Hospitals, Saveetha Institute of Medical and Technical Sciences, Saveetha University, Chennai, India; 5 Private Practice, Luxembourg, Luxembourg; 6 Biomaterials Unit, Cranio-Facial Research Laboratory, Faculty of Dental Medicine, Saint Joseph University of Beirut, Beirut, Lebanon; 7 Pôle de Médecine et Chirurgie Bucco-Dentaire, Hôpital Civil, Hôpitaux Universitaire de Strasbourg, Strasbourg, France

**Keywords:** minimal preparation, conservative, sonic activation, polymer needle, metallic needle, smear layer removal

## Abstract

**Objectives** The aim of this study was to determine the effect of apical preparation size and preparation taper on smear layer removal using a metallic needle and a new polymer needle (IrriFlex, Produits Dentaires SA “PD,” Vevey, Switzerland).

**Materials and Methods** One hundred and eight single-rooted teeth with one canal were randomly divided into four groups according to the preparation and irrigation needle used: G1—30, 0.04 and IrriFlex ( *n* = 25); G2—25, 0.06 and IrriFlex ( *n* = 25); G3–30, 0.04 and metallic needle ( *n* = 25); and G4—25, 0.06 and metallic needle ( *n* = 25). All groups received the same final irrigation protocol and sonic activation. Each tooth was sectioned and observed under a scanning electron microscope (SEM).

**Statistical Analysis** Data were statistically analyzed by using one-way and two-way analysis of variance on ranks with a significance level at *p* = 0.05.

**Results** For all groups, there was significantly higher smear layer in the apical third ( *p* < 0.001) compared with the coronal and middle thirds. The 25, 0.06 preparation demonstrated better cleaning efficiency than the 30, 0.04 preparation throughout the canal when irrigated with a metallic needle; however, there were no significant differences in the middle and apical thirds when IrriFlex was used. There were also no differences of smear layer removal between G1 and G3 and G2 and G4 in the coronal part. In the middle and apical parts, G1 showed better elimination of smear layer compared with G3. There were slight differences in the middle third between G2 and G4, while G2 showed less cleaning efficiency compared with G4 in the apical third ( *p* = 0.022).

**Conclusion** All groups showed less smear layer in the middle and coronal thirds of the canal compared with the apical third. The 25, 0.06 preparation was more effective in removing smear layer compared with the 30, 0.04 preparation. IrriFlex improved irrigation in the 30, 0.04 preparation, while its efficacy was less evident in the 25, 0.06 preparation.

## Introduction

Chemo-mechanical preparation is essential for the success of endodontic treatment. 1 Mechanical instrumentation enables the elimination of microbial biofilms and vital and/or necrotic tissue. 1 2 Nonetheless, this instrumentation leads to the accumulation of debris and the creation of smear layer. 3 The latter may act as a barrier, hindering the proper penetration of intracanal medicaments and irrigants into the dentin tubules. 4 5

Some areas of the canal remain untouched by mechanical instrumentation due to the intricate anatomy of the root canal system. 6 7 A proper chemical disinfection is necessary during endodontic treatment, especially with the anatomical complexities such as isthmuses, ramifications, apical deltas, and accessory canals. 8 Therefore, chemical irrigation that engages the bactericidal properties of sodium hypochlorite (NaOCl) and the chelating effects of ethylenediaminetetraacetic acid (EDTA) aims to diminish the smear layer, debris/tissue remnants of the untreated canal walls, and microbial presence, thereby improving canal cleanliness. 5 7 9 10 Various methods have been developed and commercialized to enhance the penetration of irrigants into deeper areas of the canal and to eliminate debris and smear layer.

A suggested method for better endodontic disinfection is increasing the canal volume. Some studies have found that increasing this volume leads to a higher capacity of the irrigant introduced into the canal, thus enhancing the treatment's effectiveness. 11 12 13 A wider taper leads to a broader storage of the irrigation solution. Subsequently, the smear layer is removed more efficiently. 11 12 14 Nonetheless, root canal enlargement reduces the tooth structure, alters the original canal anatomy, and may increase the risk of root fracture. 15 16

Minimally invasive endodontics (MIE) was recently introduced with the aim of preserving tooth structure. As a result, less tapered instruments were recommended to preserve coronal and peri-cervical dentin. 17 Nonetheless, the disinfection of minimally prepared canals can be challenging due to the limited penetration of the irrigation needle. 16 17 The effectiveness of irrigant penetration is influenced by both the anatomy of the root canal system and the final canal taper. 14 It was reported that increased canal taper favors the penetration of the irrigation needle closer to the working length (WL). 14

In clinical practice, a needle-and-syringe system is commonly employed to deliver the solution deep into the root canal. 18 19 Present research suggests that factors such as the size and design of the needle (open-ended, side-vented needle, double-side vented), the insertion depth of the needle's tip, and the flow rate of the irrigants influence the efficacy of the root canal disinfection and the removal of bacterial biofilms. 19

A new flexible polyethylene needle, IrriFlex (Produits Dentaires SA “PD,” Vevey, Switzerland), has been introduced to the market to allow, according to the manufacturer, a better penetration into the canal, a lower risk of breakage, and minimization of apical extrusion. This needle has two lateral vents and a closed end. This double-sided vented needle allows the production of two jets oriented in an oblique direction. Moreover, it delivers a large volume of irrigant at a high flow rate with less risk of apical extrusion. 19 The body's flexibility enables the needle to reach the apical region smoothly, avoiding resistance or harm to the dentinal walls. The 4% taper shape of the needle adapts to the canal's shape, ensuring a consistent thickness of irrigant flow as it progresses toward the coronal area. This consistency optimizes shear forces, promoting the removal of debris, smear layer, and biofilm. 20 21

Significant emphasis is placed on investigating the potential of modern irrigation strategies to enhance canal cleanliness, especially in cases of minimal canal preparation. Furthermore, areas within this field lacking in research are highlighted. 22 To date, despite numerous studies examining various instrumentation methods and techniques for root canal cleanliness, there are insufficient published data explaining the effect of conservative preparations on irrigation, 22 as well as the effectiveness of the IrriFlex needle compared with metallic needles regarding the removal of dentinal debris and smear layer. 19

Therefore, the aim of this study was to determine, using the scanning electron microscope (SEM), the effect of apical preparation size and preparation taper on smear layer removal using a metallic needle and a new polymer needle (IrriFlex). The first null hypothesis tested is that there is no difference between a 30, 0.04 and a 25, 0.06 preparation on the elimination of the smear layer. The second null hypothesis is that the type of irrigation needle used has no influence on smear layer removal.

## Materials and Methods

The study protocol was validated by the ethics and teaching committee of Saint Joseph University of Beirut (2023/38). To determine the sample size, power analysis using G*Power 3.1.9.7 software for Windows (Heinrich Heine, Universitat Düsseldorf, Düsseldorf, Germany) was performed for one-way analysis of variance (ANOVA). A power of 0.8 and an *α* level of 0.05 were considered. The minimum sample size required for parametric test was 76 canals (19 canals per group).

### Teeth Preselection

One hundred and eight permanent, mature, noncarious, and intact single-rooted teeth were collected for the study. The selected teeth were cleaned with an ultrasonic tip (Mectron SpA, Loreto, Italy) and preserved in 0.1% formocresol. Radiographs (Dürr Dental, Bietigheim-Bissingen, Germany) in the buccolingual direction were taken for all these teeth to ensure they present a single straight canal (≤20 degrees according to the Schneider method 23 ), showing neither calcifications nor internal resorption. Teeth with root fractures or cracks, external resorptions, and very wide canals/foramen were excluded.

### Final Teeth Selection and Root Canal Volumes

**Fig. 1:**
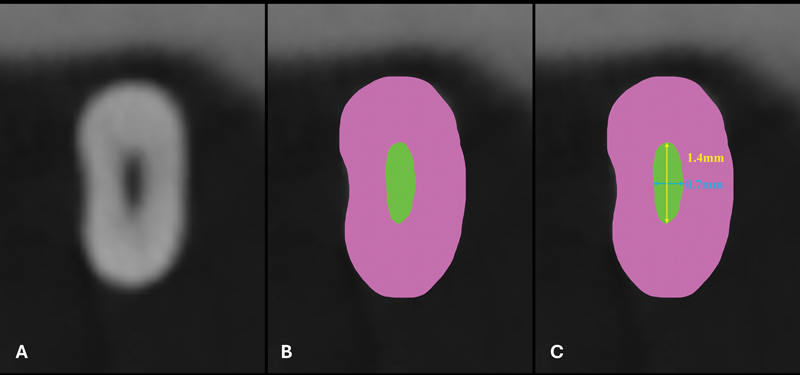
Example of an axial cross-section, 5 mm from the apex, to determine whether the canal ( *green* ) was oval in the buccolingual direction. ( **A** ) Cone beam computed tomography (CBCT). ( **B** ) Figure based on CBCT. ( **C** ) Figure with values.

**Fig. 2:**
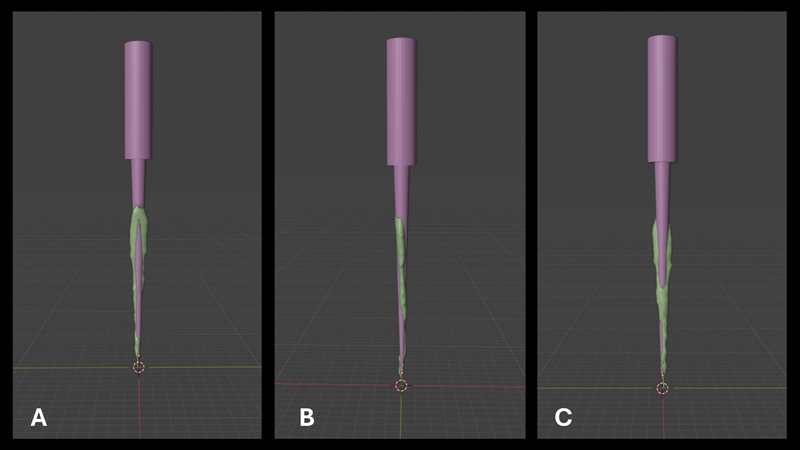
Superimposition of the instrument ( *purple* ) onto the canal ( *green* ) ensuring that the canal is encompassed by the instrument along its majority length. ( **A** ) Distal. ( **B** ) Buccal. ( **C** ) Mesial.

A cone beam computed tomography (CBCT; Sirona, Bensheim, Germany) was performed on the preselected teeth to calculate the root canal volume and ensure the following:

- The canals were comparable. The canal volume averaged 5.84 ± 0.93 mm ^3^ .
- The canals were classified as oval, on an axial cross-section, when the buccolingual diameter, 5 mm from the apex, was at least two times wider than the mesiodistal diameter (in millimeter; Fig. 1 ). 24
- The two R-Motion files used (30, 0.04 and 25, 0.06) had a caliber larger than the canal diameter in the mesiodistal direction ( Fig. 2 ) and the initial foramen's diameter of the canals was less than 0.25 mm.

The three-dimensional (3D) design of the instruments 30, 0.04 and 25, 0.06 was first performed on Blender software (version 3.5), while the segmentation of the tooth and canal was made on 3D Slicer (version 5.2.2). The segmentation of the canal was exported to an STL file that was then imported on Blender to align the instrument in the canal and compare the mesiodistal diameters ( Fig. 2 ).

### Access Cavity and Root Canal Standardization

The entire procedure was performed by a single operator to avoid interoperator errors. An access cavity was made on all teeth using an 802-diamond bur (Komet Italia SRL, Milan, Italy) mounted on a high-speed handpiece under running water. The ceiling was removed using a Zekrya Endo-Z bur (Dentsply Sirona). The canal was scouted with K-prep (REKITA, Lebanese Dental Products) and a no. 10 K-file (Dentsply Sirona), until reaching the foramen and ensuring patency. The crowns were sectioned with a diamond disk (Kerr Dental, Bioggio, Switzerland) to standardize the root length at 18 mm (± 1 mm). A reservoir in the pulp chamber was kept to standardize the conditions of irrigation. The WL was established at –1 mm from the foramen using a no. 15 K-file (Dentsply Sirona). The WL was therefore 17 mm (± 1 mm).

### Root Canal Preparation and Irrigation

All the selected teeth had a straight canal, a foramen diameter ≤0.25 mm, same length, and comparable canal volumes. The teeth were then randomly divided into four main groups ( *n* = 25) and a control group ( *n* = 8) following the shaping instruments and irrigation needles:

- **Group 1:** The glide path was ensured with the R-Motion glider (FKG Dentaire) at the WL with an in-and-out motion. Irrigation of 3 mL of 6% NaOCl with the IrriFlex needle was performed, followed by a recapitulation with the no. 10 K-file at WL. The needle was inserted 1 mm from its flexion point on the walls and not more than 1 mm from the WL. Then, the crown down was performed with the R-Motion (30, 0.04) on a Rooter Universal motor (FKG Dentaire) with a reciprocating motion pre-established by the manufacturer. Each instrument was used for the preparation of a single canal, in the coronal part, then in the middle part, and finally in the apical part with an in-and-out motion and an amplitude of approximately 3 mm. Between each use, the instrument was removed, cleaned, and inspected. The canal was irrigated with 3 mL of 6% NaOCl, then a no. 10 K-file was introduced to maintain the apical patency.
- **Group 2:** The protocol of this group was similar to that of group 1, but the preparation was performed with an R-Motion (25, 0.06).
- **Group 3:** The protocol of this group was similar to that of group 1, but the irrigation was performed with a 30-gauge (closed with double-side vented) metallic needle (C-Kendo, C-K Dental, Korea).
- **Group 4:** The protocol of this group is similar to that of group 2, but the irrigation was executed with a metallic needle.
- **Control group:** Each pair of teeth in this group underwent the same protocol as groups 1, 2, 3, and 4, but without the following step of final irrigation.

### Final Irrigation

At the end of shaping, the apices were covered with wax to ensure a closed system and the final irrigation was performed as follows 5 : 2.5 mL of NaCl (saline water) at 0.9% for 90 ± 5 seconds was applied. After aspiration and drying, 5 mL of 17% EDTA over 120 ± 10 seconds was injected into the canal, followed by 1 minute of sonic activation using EQ-S cordless sonic endo irrigator (Meta Biomed, Chungcheongbuk-do, South Korea). When the EDTA was eliminated, 2.5 mL of 0.9% NaCl for 90 ± 5 seconds was injected, then 5 mL of 6% NaOCl over 120 ± 10 seconds was followed by 30 seconds of EQ-S activation, aspiration, and drying. A final rinsing of 2.5 mL of 0.9% NaCl for 90 ± 5 seconds was executed. In all cases, the needle was introduced at –1 mm from the established WL, therefore at 16 (± 1) mm, with light in-and-out movements and an amplitude of 1 to 2 mm. The activation was executed with a tip of 25/02 at 13,000 cycles/min (217 Hz). 5 25 The tip was introduced at 16 (± 1) mm and the activation was performed with an in-and-out movement of 3- to 4-mm amplitude.

### Tooth Preparation for Scanning Electron Microscope

The roots were carefully cut into six pieces using a diamond disk (Kerr Dental). The tooth was first cut, perpendicularly to the longitudinal axis, into three segments of 5 (± 1) mm each: coronal, middle, and apical. Each third was then divided into two longitudinal fragments in the buccolingual direction to observe the internal dentinal walls of the root canal. Buccal and palatal longitudinal grooves were formed without penetrating the canal to avoid any displacement or addition of debris during the sectioning procedure. A chisel and a mallet were used to split each sample. 25 After that, all the specimens were dehydrated in a graded series of ethanol solutions (50, 70, 90, and 100%, 10 minutes each). After drying, the samples were mounted on SEM stubs and sputter coated with a gold–palladium alloy (20/80 weight %) using a HUMMER JR sputtering device (Technics, San Jose, CA, United States). The coated specimens were finally analyzed using the Quanta 250 FEG SEM (FEI Company, Eindhoven, The Netherlands) with an electron acceleration voltage of 10 kV to evaluate the amount of remaining smear layer at the coronal, middle, and apical third of all the specimens of each group.

### Scanning Electron Microscope Observation

One SEM micrograph at ×2,000 magnification, showing the canal wall surface in the area containing the greatest amount of smear layer, was taken from the coronal, middle, and apical thirds of each tooth. In total, 300 micrographs were analyzed and then classified according to the grading system of the remaining smear layer. 26

**Fig. 3:**
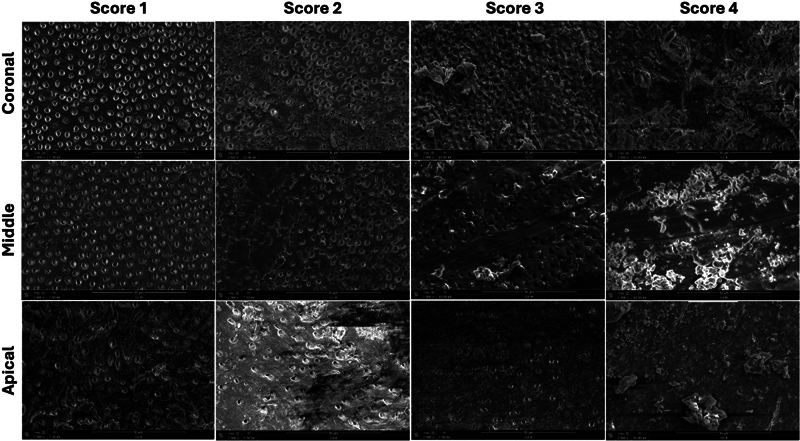
Representative scanning electron microscopy micrographs (×2,000) of each smear layer score in the coronal, middle, and apical thirds of root canals.

The classification of Gutmann et al 26 was applied to all the micrographs. Four scores were used for grading the micrographs ( Fig. 3 ):

- Score 1: Little or no remaining smear layer, covering less than 25% of the specimen with visible and patent dentinal tubules.
- Score 2: Little to moderate or patchy amount of smear layer, covering between 25 and 50% of the specimen with many visible and patent dentinal tubules.
- Score 3: Moderate amounts of scattered or aggregated smear layer, covering between 50 and 75% of the specimen with minimal to no visible or patent dentinal tubules.
- Score 4: Heavy smear layer present, covering more than 75% of the specimens with no visible or patent tubule orifices.

Each micrograph was analyzed by three blinded experienced SEM examiners. In case of disagreement, a discussion has been initiated to reach a compromise.

### Statistical Analysis

One-way ANOVA was used to compare the smear layer removal efficacy between the three segments in each group. Two-way ANOVA on ranks was also used to compare the effectiveness of smear layer removal among the four groups. Data analysis was performed with Sigma Plot (11.2, Systat Software, Inc., San Jose, CA, United States). A significance level of *α* = 0.05 was adopted. Cohen's kappa test was applied to verify the agreement between the two observers using Minitab software (Minitab 18.1, Minitab, Inc., Pennsylvania State University, PA, United States).

**Fig. 4:**
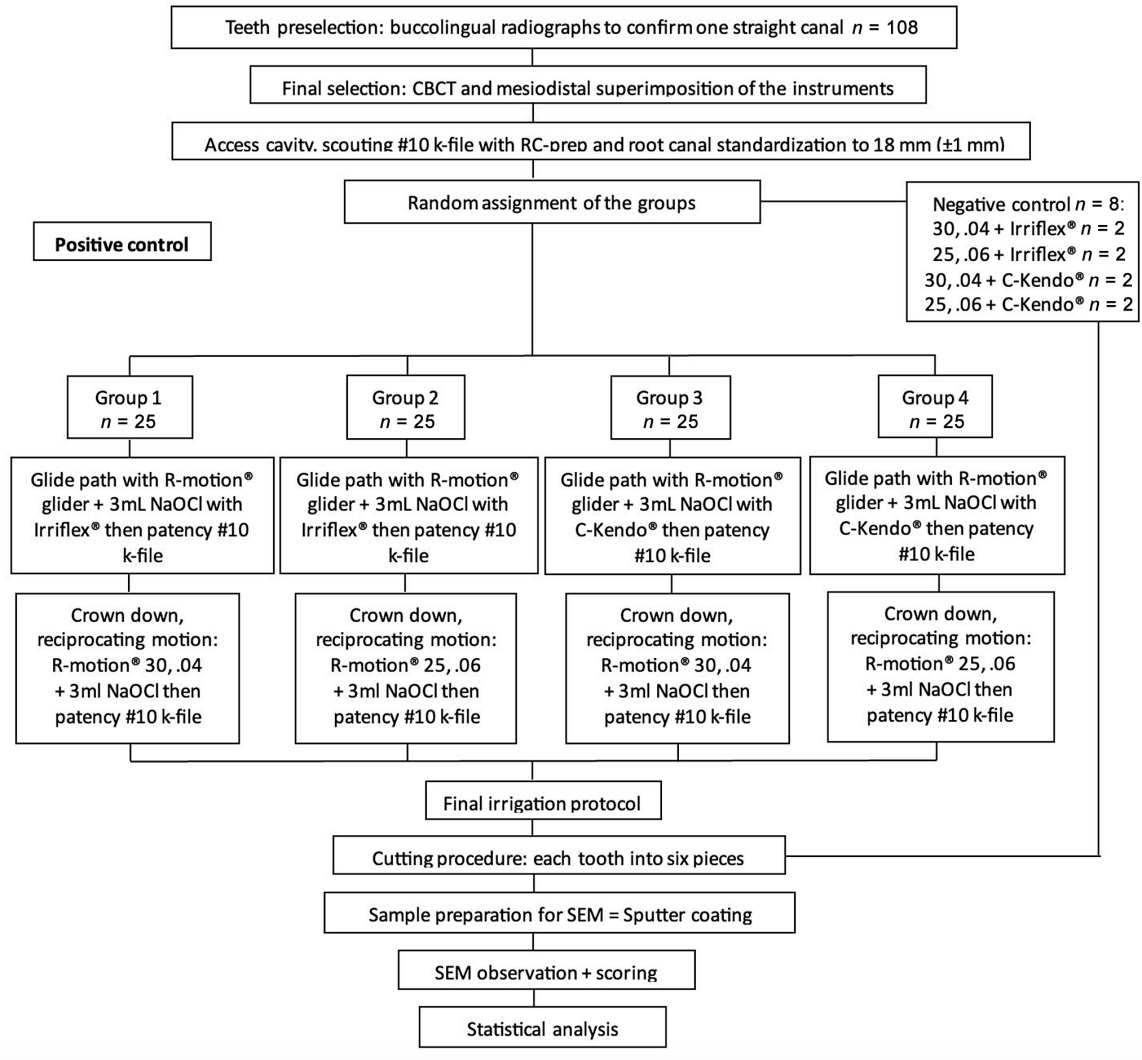
Flowchart of the methodology. CBCT, cone beam computed tomography; SEM, scanning electron microscope.

The study design has been summarized in a flowchart in Fig. 4 .

## Results

The Cohen's kappa value for interobserver agreement of all groups and subgroups was 0.89.

The comparison of smear layer removal among the three segments in each group was performed by one-way ANOVA. In all groups, the apical third demonstrated lower smear layer removal compared with the middle and coronal thirds ( *p* < 0.001). However, no significant differences were found between the coronal and middle thirds ( *p* > 0.05; Table 1 ).

**Table 1:** Mean and standard deviations of the reported scores of each group

	G1	G2	G3	G4
Coronal	1.64 ± 0.63 ^b^	1.12 ± 0.33 ^b^	1.44 ± 0.71 ^b^	1.04 ± 0.2 ^b^
Middle	1.32 ± 0.55 ^b^	1.36 ± 0.56 ^b^	1.76 ± 0.87 ^b^	1.24 ± 0.66 ^b^
Apical	2.6 ± 0.8 ^a^	2.36 ± 0.95 ^a^	3.12 ± 0.66 ^a^	1.64 ± 0.63 ^a^
*p* < 0.05	a < b *p* < 0.001	a < b *p* < 0.001	a < b *p* < 0.001	a < b *p* < 0.001

Note: Significant differences are denoted by superscript a and b.

Two-way ANOVA was used to investigate the effect of taper and material on smear layer removal.

Concerning the comparison of the four groups in the coronal third, the difference in the mean values of material is not significant enough to have a statistically significant difference ( *p* = 0.178). Two-way ANOVA demonstrated a significant difference between the 25, 0.06 and the 30, 0.04 preparations ( *p* < 0.001). Therefore, a significantly higher smear layer removal effect of the 25, 0.06 preparation was found compared with the 30, 0.04 preparation.

At the middle third, while comparing the four groups, no significant difference was found between the 25, 0.06 and the 30, 0.04 preparations for the IrriFlex needle ( *p* = 0.836). Concerning the metallic group, the 25, 0.06 preparation demonstrated a higher effect compared with the 30, 0.04 preparation ( *p* = 0.008). Regarding the 25, 0.06 preparation, there was no significant difference between the IrriFlex and metallic needles ( *p* = 0.534). However, the 30, 0.04 preparation demonstrated a significantly higher effect while using the IrriFlex needle as opposed to the metallic one ( *p* = 0.024).

Concerning the apical third, there was a statistically significant difference between taper and material. No significant difference was found between the 25, 0.06 and 30, 0.04 preparations for the IrriFlex needle ( *p* = 0.286). As for the metallic group, the 25, 0.06 preparation demonstrated higher effectiveness in contrast to the 30, 0.04 preparation ( *p* < 0.001). A significantly higher effect was observed with the metallic needle compared with the IrriFlex needle ( *p* = 0.002). Nonetheless, the 30, 0.04 preparation demonstrated a significantly higher effect of the IrriFlex needle compared with the metallic one ( *p* = 0.022).

All the distribution of scores in each third of each group is shown in Table 2 .

**Table 2:** Score percentages of each third in each group

Third	Scores	G1 (%)	G2 (%)	G3 (%)	G4 (%)
Coronal	1	44	88	68	96
	2	48	12	20	4
	3	8	0	12	0
	4	0	0	0	0
Middle	1	72	68	48	84
	2	24	28	32	12
	3	4	4	16	0
	4	0	0	4	4
Apical	1	8	16	0	44
	2	40	48	16	48
	3	36	20	56	8
	4	16	16	28	0

All the results are presented in Table 3 .

**Table 3:** Results of the effect of taper and material on smear layer removal

	Taper	Material
Coronal	G1 < G2 G3 < G4	G1 = G3 G2 = G4
Middle	G1 = G2 G3 < G4	G1 > G3 G2 = G4
Apical	G1 = G2 G3 < G4	G1 > G3 G2 < G4

## Discussion

The aim of this study was to determine the effect of apical preparation size and preparation taper on smear layer removal using a metallic needle and a new polymer needle (IrriFlex).

The first null hypothesis was rejected because a notable difference was observed between the final tapers in the removal of smear layer. The second null hypothesis was also rejected because the elimination of smear layer was affected by the type of the irrigation needle ( *p* < 0.05).

Comparing the three segments of each group, results showed that the apical third exhibits higher score values than both coronal and middle thirds. This finding reproduces similar results found in other comparable studies. 27 28 The apical portion is the narrowest part of the canal and thus has the most reduced canal diameter. 27 28 The diameter of the canal influences both the efficiency of debris removal 29 and the volume and exchange of irrigant at the WL. 8 Subsequently, cleaning the apical third is found more challenging across all groups. Moreover, the activation technique used in this protocol can also explain these differences. Research has found that ultrasonic activation promotes significantly more irrigant penetration than sonic activation. 30 The limited acoustic cavitation effect generated by sonic devices may affect the penetration of irrigants 31 32 and hence the cleaning efficiency. The EQ-S, used in our study, is a sonic activation device that possibly resulted in less effective outcome in the apical third.

Clinically, during the root canal treatment, the mesiodistal direction of a canal is visualized on a periapical radiograph. The canal appears thin and may coincide with the instrument's position halfway along the canal on a CBCT scan. However, the canal is oval in the buccolingual direction and is much wider than the instrument. The file is rather circular and intended for use in the middle of oval canals. Hence, substantial portions of oval canal walls are not involved during the mechanical instrumentation of the root canal. 7 This could explain why some areas of the canal sections showed more smear layer than others.

At the coronal third, among the four groups, two-way ANOVA test demonstrated a significant difference concerning the effect of taper size on the smear layer removal. G2 (25, 0.06: IrriFlex) and G4 (25, 0.06: C-Kendo) have a taper of 6%, while G1 (30, 0.04: IrriFlex) and G3 (30, 0.04: C-Kendo®) have a taper of 4%. It is believed that the shaping with bigger taper results in greater removal of dentin from the canal walls, thus producing a cleaner root canal system. 12 19 As a result, G2 and G4 had, respectively, more cleaning efficiency than G1 and G3 in the coronal part.

At the middle and apical thirds, among G1 and G2, no significant difference was found between the 25, 0.06 and the 30, 0.04 preparations with IrriFlex irrigation. Thus, the taper had no effect when IrriFlex was used. At the apical third, almost the same amount of dentin was removed in both tapers, D _5_ (30, 0.04) = 0.5 and D _5_ (25, 0.06) = 0.55. In addition, the volumes of the apical part of the used instruments, 30, 0.04 and 25, 0.06, have respectively close volumes of V(30, 0.04) = 0.64 mm ^3^ and V(25, 0.06) = 0.66 mm ^3^ . Furthermore, all canal volumes were comparable within a margin of 5.84 ± 0.93 mm ^3^ , and therefore, the volume of irrigant in the apical part prepared by both instruments was roughly the same. Additionally, the same tapered needle was used for the same time for the two groups. Accordingly, tapered needles enhance irrigation efficiency by directing the flow of irrigant toward the apex and improving penetration into canal irregularities. This explains the lack of significant differences between G1 and G2 in the apical and middle parts.

In contrast, the same conditions were applied in G3 and G4, but the results showed a dissimilarity. G4 demonstrated higher efficacy in the removal of smear layer compared with G3 in the apical and middle thirds. This could be explained by the design of the needle. While IrriFlex is a 4% tapered needle, C-Kendo is a cylindrical one. The cylindrical design of C-Kendo is less effective in directing irrigant toward the apex or accessing narrow and curved areas of the root canal system. It may promote a more even distribution of irrigants throughout the canal. Additionally, the whole canal volume prepared with the 25, 0.06 file is larger than the canal prepared by the 30, 0.04 file, according to the same concept above. The files used, 25, 0.06 and 30, 0.04, have, respectively, a volume of V _1_ = 7.662 mm ^3^ and V _2_ = 5.259 mm ^3^ . Therefore, there is a larger volume of irrigation solution in the canal prepared to 25, 0.06. In fact, Brunson et al confirmed Wandelt's statement, which demonstrated that the larger the canal volume, the greater the volume of the irrigant, and the better the canal cleaning will be performed. 12 13 It has also been reported by Albrecht et al that increasing the taper of the root canal has a direct effect on irrigant flow, resulting in improved debridement during irrigation. 33 Moreover, a greater taper dislodges and removes debris more efficiently. This explains the significant difference between G3 and G4 in the coronal, middle, and apical parts, with a better elimination of smear layer in G4 in the three segments.

The concept of apical dimension and canal taper has been extensively investigated in the literature with contradictory results. Zarei et al explained that for the same apical dimension of 30, the increase in taper did not influence the quantity of smear layer in the apical third. 16 34 Plotino et al confirmed this assertion by showing that for the same apical dimension, a taper of 6% did not eliminate a greater quantity of smear layer compared with a preparation of 4% in the apical third. 16 In contrast, van der Sluis et al observed that taper had a direct correlation with canal debridement. 29 34 The possible explanation for these results may be based on root selection. The selected roots in this study were single straight canals, and in the van der Sluis et al study, the selected teeth were mandibular and maxillary canines, while Zarei et al selected molar roots. Consequently, the more the canal is curved, the less the penetration of the irrigant is affected by the taper of the canal. Moreover, the difference in shaping instrument, shaping motion (brushing, pecking or in-and-out motion, etc.), irrigation and activation protocols, and devices could explain the diverse results.

In the apical third, Plotino et al reported that a basic preparation of 25, 0.06 produced significantly less clean root canal walls than a 40, 0.04 preparation. 16 35 Similarly, Xu et al proved that the amount of debris was significantly less with a 40, 0.04 preparation compared with 25, 0.04; 30, 0.04, and 35, 0.04 preparations. 36 This explains that the small difference between the foramen diameter of this study, 25 and 30, did not enhance the smear layer removal; however, while starting by a foramen of 40, the irrigation has been improved compared with a foramen of 25.

The enlargement of the foramen diameter to 30, in this study, did not optimize the elimination of smear layer no matter the type of the needle. Therefore, it is better not to enlarge the foramen to prevent apical laceration. This reminds us of Schilder's mechanical objectives of root canal preparation: apical foramen should remain in its original spatial relationship and should be kept as small as practical in all cases. 22 Removing the smear layer is strongly correlated with the design of the needle and the entire canal space above the foramen, rather than merely the diameter of the foramen. Consequently, the irrigation of a basic preparation of 6% and a minimal preparation of 4% did not give the same results regarding the elimination of the smear layer in the coronal, middle, and apical thirds of the canal.

The two needles used were placed, during the final irrigation, at –1mm from the WL of the straight canals, without curvatures and therefore without obstacles. By analogy, for a preparation of 25, 0.06 and a preparation of 30, 0.04, at –1 mm from the WL, there was a dimension of 0.31 and 0.34 mm, respectively. In addition, the two needles had a diameter of 30 gauge, which is equivalent to 0.31 mm, and two side exits, which mean that all the needles reach the WL of –1 mm to eliminate the same quantity of the smear layer with double-side vented. However, their efficiency was different due to the body design of the needles.

The size of the IrriFlex needle and the final shaping of G1 and G3 were all 30, 04. Indeed, the needle fitted snugly into the canal while directing the irrigant apically, and therefore maximized the shear forces and the elimination of smear layer. Moreover, by maintaining a constant flow thickness, the shear forces generated during irrigation were maximized, leading to more effective elimination of debris, smear layer, and biofilm from the root canal system. 20 21 However, this snug fit, especially coronally, may create a seal and restrict fluid exchange to some extent. Consequently, for a 30, 0.04 preparation, IrriFlex has a better cleaning effect in the apical and middle parts compared with the metallic needle, while they have the same efficiency in the coronal third.

The final preparation of G2 and G4 was 25, 0.06; thus, both needles were looser in the canal. According to Boutsioukis et al, the space available around the needle is crucial for facilitating the backward flow of irrigant toward the canal opening, significantly affecting the overall effectiveness of irrigation. 14 Consequently, the IrriFlex and C-kendo needles showed no significant differences in the coronal and middle thirds. Concerning the apical third, the metallic needle had a better cleaning efficiency than the IrriFlex needle. Indeed, only straight canals were included in this study. Therefore, in straight root canals, fluid exchange and replacement occur relatively straightforwardly due to the absence of significant anatomical complexities or obstacles. The irrigant flows in a relatively linear path from the access cavity to the apex, facilitating efficient cleaning and disinfection along the entire length of the canal.

In fact, while tapered needles can still be advantageous in straight root canals by providing effective sealing and shear forces, their superiority over other needle designs may not be as significant as in thin or complex-shaped canals. Straight canals may not require the same level of adaptation and flexibility provided by tapered needles, since they lack the challenges associated with curved or thin canals. Other needle designs such as double-side vented offer comparable or even superior performance in straight canals.

To the best of our knowledge, data on IrriFlex are still scarce and minimal. According to Hussein et al, IrriFlex was more effective than ProRinse in removing smear layer with EDTA and citric acid. 21 These results can be attributed to the different types of needles used: ProRinse is a side-vented needle and IrriFlex is a double-sided one. Further studies are therefore needed for a wholesome understanding of the intricacies of the IrriFlex needle.

It is important to note that numerous studies have long explored methods to improve irrigation protocols. However, these studies have yielded varied results, which may be attributed to differences in the geometry and design of the instruments used, as well as variations in irrigation protocols. These variations include differences in irrigant solutions, temperature, the presence or absence of surfactants, activation methods, duration of activation, and final preparation size, including taper and apical diameter. 37 From Mancini et al's study to the present, no research has demonstrated complete elimination of the smear layer. 37 38 39 Current dental knowledge does not identify a single optimal root canal irrigation protocol that guarantees clinical success in endodontic treatment. 37

Despite the novelty of the study, it is essential to acknowledge its limitations. All the teeth included in this study were straight canals without curvatures. Two samples from different teeth were lost during the cutting process and they were subsequently replaced. This study was conducted under ideal, *in vitro* conditions, which differ from clinical settings and thus limits its clinical impact. Microcomputed tomography (µCT) should be used to measure canal volume and to evaluate smear layer removal. During SEM preparations, some invasive steps such as cutting could generate supplementary debris into the root canal system. µCT offers high resolution and a nondestructive approach, making it advantageous for *in vitro* research compared with traditional methods. Sonic activation was performed by EQ-S (Meta Biomed) for all the groups; thus, further research on other devices should be performed. The microscope allows the evaluation of a localized part from the entire surface of the third concerned. To overcome this limitation, the entire surface was fully scanned and the area with the greatest amount of smear layer was taken and analyzed. Digital images are two-dimensional images, and therefore the thickness of the smear layer cannot be quantified. Debris and smear layer removal are best studied together; however, this study was conducted using only high magnification. Additional investigations should use µCT to examine the effects of various instrument sizes and tapers on the dentin volume removal and smear layer removal.

Further studies should be applied on the effect of IrriFlex and minimal preparations on curved or **S** -shaped canals and in the reduction of smear layer, debris, and/or biofilm. More extensive *in vivo* studies can be warranted to assess whether the minimal preparations enhance tooth longevity and promote the healing of pulp pathologies.

## Conclusion

Within the limitations of this study, the 25, 0.06 preparation eliminated smear layer more efficiently than the 30, 0.04 preparation. Moreover, IrriFlex offered better irrigation in conservative canals. There was significantly less smear layer in the coronal and middle thirds of the canal compared with the apical third, with no differences among the groups.
